# Non-HFE Hemochromatosis in the Context of β-Thalassemia Trait: A Case Study on Iron Overload Dysregulation

**DOI:** 10.7759/cureus.74431

**Published:** 2024-11-25

**Authors:** Kate Jensen, Andrew Hammer, Ahmad Khatib, Moustafa Hazin

**Affiliations:** 1 Medicine, Creighton University School of Medicine, St. Joseph’s Hospital and Medical Center, Phoenix, USA; 2 Internal Medicine, Creighton University School of Medicine, St. Joseph’s Hospital and Medical Center, Phoenix, USA

**Keywords:** beta-thalassemia minor, hemochromatosis, hereditary hemochromatosis (hh), iron overloaded, non-transfusional hemochromatosis, non-transfusion-dependent thalassemia, thalassemia trait

## Abstract

Thalassemia and hemochromatosis are two distinct conditions that involve dysregulation of iron metabolism, though their origin, clinical presentations, and treatments differ. This case represents a patient with incidentally discovered microcytic anemia due to β-thalassemia trait and non-*HFE* hemochromatosis. It discusses the potential synergistic effect of these two diseases on iron overload and highlights the need for further testing to determine hereditary versus secondary causes of hemochromatosis. In addition, this case study also offers insight into the management of these conditions with somewhat conflicting treatments. In this case, the patient was advised to avoid phlebotomies so as not to worsen the anemia and was referred to hepatology.

## Introduction

β-thalassemia is among the most common autosomal recessive disorders caused by either a reduced or absent production of β-globin chains of hemoglobin tetramer. This can typically be identified in a patient with microcytic anemia characterized by a decreased mean corpuscular volume and confirmed with hemoglobin electrophoresis showing increased amounts of hemoglobin A2 (HbA2) and hemoglobin F (HbF) [1,2]. β-thalassemia clinically presents in three ways, namely, carrier state, thalassemia intermedia, and thalassemia major. Patients with the carrier state with the β-thalassemia trait leading to a partial deficiency in the β-globin chains of hemoglobin are typically asymptomatic and their anemia is discovered on routine blood work. Most do not require any treatment [1].

For symptomatic major β-thalassemia patients, substantial medical intervention is typically necessary for the anemia in the form of blood transfusions. Chronic transfusions, however, can lead to complications such as iron overload. However, even in patients who are not receiving transfusions, β-thalassemia intermedia and major patients are at risk for iron overload potentially due to decreased hepcidin hormone, ineffective erythropoiesis, and inflammation of the gastrointestinal (GI) tract affecting iron absorption [3-5].

Hemochromatosis is a condition of iron overload in which organs accumulate excessive iron leading to end-organ damage. The most common cause is hereditary with a mutation of the *HFE* gene responsible for the hepcidin hormone that controls iron absorption in the GI tract [6]. However, in some cases, etiology stems from non-hereditary (secondary) factors and further tests are necessary to identify the cause [5,7,8]. Other causes include increased intake of iron from diet or repeated blood transfusions such as in thalassemia patients or chronic liver disease such as cirrhosis [6]. The treatment for hemochromatosis involves routine phlebotomy, which reduces iron stores by removing blood, or iron chelation therapy, which binds excess iron and facilitates its excretion through the kidneys [9]. However, the risks of this include iron deficiency anemia, hypovolemia, infection, and electrolyte imbalances [9].

## Case presentation

The patient was a Hispanic male with no significant past medical history who presented to a free clinic for a routine checkup. Upon routine blood work, he was found to have microcytic anemia with a hemoglobin of 12.7 g/dL, a mean corpuscular volume of 62 fL, and a red blood cell distribution width of 21%. This prompted further workup to classify the anemia. Folate and B12 were normal. Haptoglobin and lactate dehydrogenase were also within normal limits, indicating the absence of hemolytic anemia.

Iron studies were largely normal with a total iron binding capacity of 285 µg/dL, an iron level of 95 µg/dL, and an iron saturation of 33%; however, the ferritin levels were exceedingly high with a value of 2,000 ng/mL. Hemoglobin electrophoresis was completed, showing elevated levels of HbA2 and a slightly high HbF, indicative of β-thalassemia trait.

Ferritin is a protein that stores iron in the body, and elevated levels can indicate iron overload, inflammation, or liver disease. This finding raised concerns for conditions such as hemochromatosis or other chronic inflammatory states. To work up the increased ferritin levels, a series of tests were completed, including antinuclear antibodies, rapid protein reagent, and hepatitis B/C, which were all negative. Erythrocyte sedimentation rate and C-reactive protein were within normal limits. Transaminases were within normal limits with aspartate transaminase at 34 U/L and alanine transaminase at 50 U/L, and platelets were 229 × 10^9^/L (Table 1). However, fibrosis staging did show mild scarring with F1-F2 levels, and total bilirubin was elevated at 2.0 mg/dL. Peripheral blood smear showed anisocytosis, microcytosis, dacrocytes, and basophilic stippling. There were no abnormalities on the physical examination.

**Table 1:** Laboratory values.

Parameter	Value	Unit
Complete blood count		
Hemoglobin	12	g/dL
Mean corpuscular volume	62	fL
Red blood cell distribution width	21	%
Vitamin and nutritional levels		
Folate	10.0	ng/mL
B12	1347	pg/mL
Iron studies		
Ferritin	2,000	ng/mL
Total iron binding capacity	285	µg/dL
Iron	95	µg/dL
Iron saturation	33	%
Hemolysis markers		
Haptoglobin	66	mg/dL
Lactate dehydrogenase	163	IU/L
Reticulocyte count	3.0	%
Liver function tests		
Aspartate transaminase	34	IU/L
Alanine transaminase	50	IU/L
Total bilirubin	2.0	mg/dL

Given some of the abnormal laboratory values, a liver biopsy was completed. The iron stain of the liver parenchyma was positive for 3+ iron deposition in the majority of hepatocytes, suggesting the diagnosis of hemochromatosis. Biopsy was negative for any copper deposition or alpha-1-antitrypsin deficiency. A hereditary hemochromatosis panel was ordered and was interestingly negative for any *HFE* gene mutations.

The treatment for this patient included education on a low iron diet and deferoxamine, and iron chelation therapy to treat the iron overload in this patient by binding to excess iron in the bloodstream. He was also referred to hepatology but was unable to follow up due to lack of insurance.

Regarding the thalassemia trait, the patient was asymptomatic but will continue to be monitored with routine blood workups and educated on the inheritance of the β-thalassemia trait.

## Discussion

Here we discuss the unique case of a patient with incidentally asymptomatic β-thalassemia trait and non-*HFE* hemochromatosis. While secondary hemochromatosis is a potential consequence of β-thalassemia major and intermedia due to dysregulated iron absorption in the GI tract or for patients requiring long-term blood transfusions, this is not typically seen in patients with the β-thalassemia trait [10,11]. In particular, this patient was asymptomatic and his thalassemia trait was only incidentally discovered during routine laboratory work.

In addition, the β-thalassemia trait in hereditary hemochromatosis individuals has been reported to exacerbate iron overloads and place patients at an increased risk for complications such as cardiomyopathy and cirrhosis [10,11]. However, this relationship has been questioned in previous studies [12]. This patient did not have any *HFE* gene mutations. However, due to clinic resource limitations, the patient was only tested for *HFE* gene mutations, not any *TFR2* or ferroportin mutations that less commonly cause hereditary hemochromatosis [13,14]. This raises the question of whether the hemochromatosis is secondary to known complications of β-thalassemia which can impair iron absorption in the gut or if it was a coincidental secondary diagnosis. Further research including more case studies is needed to make conclusions on this relationship.

Regardless, this case underscores the potential interplay between these two disorders in the absence of traditional risk factors for iron overload. It also shows how careful consideration is needed to manage these coexisting conditions with more routine monitoring of hemoglobin and reducing the iron in the body without phlebotomy; however, individualized treatment plans should be formed to avoid potential complications. In asymptomatic carriers, alternative therapies such as low-dose iron chelation agents, such as deferoxamine, or adjustments to dietary intake, including limiting iron-rich or fortified foods, such as red meats and various cereals, may be considered [13,14]. These measures could offer a non-invasive approach to managing mild iron overload while minimizing the risk of disrupting the patient’s overall health.

Lastly, this case highlights the systemic issues in the healthcare system of the United States in which the uninsured are unable to be seen by specialists for their medical conditions due to lack of insurance and inability to afford necessary treatments. This gap in care may lead to delays in diagnosis and management, which may worsen patient outcomes in cases like this one.

## Conclusions

This case highlights a rare instance of a patient with incidentally diagnosed non-*HFE* hemochromatosis and β-thalassemia trait. It underscores the complexity of diagnosing and managing iron overload in patients where these two conditions coexist, as the β-thalassemia trait typically does not result in significant iron dysregulation, unlike more severe forms of thalassemia. It also highlights important considerations regarding the mechanisms of iron metabolism and the appropriate therapeutic approach, balancing the risks of iron overload against the potential complications of aggressive therapies like phlebotomy such as infection and anemia. In addition, this case adds to the literature on non-*HFE* hemochromatosis, offering insights into how it may interact with underlying genetic conditions such as the β-thalassemia trait.
